# Simultaneous Bladder Drainage via Suprapubic and Urethral Catheters: Which Drains More Completely and Why?

**DOI:** 10.26502/jsr.10020316

**Published:** 2023-09-19

**Authors:** Aurash Naser-Tavakolian, John M Masterson, Jeremiah Dallmer, Catherine Bresee, Michael Zaliznyak, Hanson Zhao, Sandeep Sandhu, Maurice M Garcia

**Affiliations:** 1 Department of Urology, Cedars-Sinai Medical Center, Los Angeles, CA, USA; 2 Biostatistics Shared Resources, Cedars-Sinai Samuel Oschin Comprehensive Cancer Center, Los Angeles, CA, USA; 3 Saint Louis University School of Medicine, Saint Louis, MO, USA; 4 Hoag Hospital, Newport Beach, CA, USA; 5 Department of Urology and Department of Anatomy, University of California San Francisco, CA, USA; 6 Cedars-Sinai Transgender Surgery and Health Program, Cedars-Sinai Medical Center, Los Angeles, CA, USA

**Keywords:** Urinary catheters, Urethroplasty, Quality improvement, Reconstructive urology, Transgender surgery

## Abstract

**Background:**

Reconstructive urologists often place both a urethral and suprapubic catheter intraoperatively to prevent extravasation of undrained urine across anastomosis sutures. As no consensus exists on which catheter drains the bladder more completely, many surgeons leave one catheter to gravity drainage and cap the other postoperatively. We sought to identify differences in catheter urine outflow during dual bladder drainage with suprapubic and urethral catheters in postoperative urology patients.

**Methods:**

Urine output (UOP) from transgender men who underwent Stage II Phalloplasty with urethral lengthening was retrospectively reviewed. Both 16 French urethral and suprapubic catheters were placed to gravity drainage postoperatively. Urine output from each catheter was recorded separately, twice daily. Mixed model regression modeling tested for differences in urine output by time of day (day/night) and activity status (Bedrest: Postop Day 0–2, Ambulatory: Postop Day 3+).

**Results:**

The aggregate number of 12-hour shift urine output observations was 250 (125 for urethral and 125 for suprapubic catheters) across 14 inpatients. Suprapubic catheters had a mean 410 ml higher output than urethral catheters per 12-hour shift (p=0.002; 95% CI: 185, 636 ml). During daytime, Suprapubic catheters demonstrated higher UOP than urethral catheters per 12-hour shift (Estimated Difference: 464 ml; p=0.002; 95% CI: 211, 718 ml). During nighttime, a similar phenomenon was observed (Estimated Difference: 356 ml; p=0.009; 95% CI: 104, 606 ml). When comparing mean UOP from each catheter during the Bedrest Phase, suprapubic catheters averaged an estimated 295 ml higher UOP compared to urethral catheters per 12-hour shift with a trend toward statistical significance (p=0.052; 95% CI −3, 594 ml). During the Ambulatory Phase, mean suprapubic catheter UOP was an estimated 472 ml higher than urethral catheters per 12-hour shift (p=0.009; 95% CI 142, 802 ml).

**Conclusions:**

Simultaneous bladder drainage with urethral and suprapubic catheters shows greater drainage from the suprapubic catheter (35% vs 65%). When using two catheters, both can be placed to gravity to maximize bladder drainage as the suprapubic catheter can drain residual urine not adequately drained by the urethral catheter.

## Introduction

The history of urinary catheterization extends back millennia into the Egyptian epoch, where evidence suggests the use of bronze tubes and reeds for bladder decompression in 1500 BC [1,2]. Since the advent of the Foley catheter as a device for continuous urinary drainage after prostatic resection in the 1930s, indwelling urinary catheters have become ubiquitous in modern medical practice [2,3]. Approximately 15% to 25% of hospitalized patients are catheterized at least once during admission [4]. While Foley catheter system drainage characteristics and complete bladder drainage are not fully understood, urethral catheters (UC) and suprapubic (SP) catheters remain essential to the postoperative care of urologic patients [5]. In the course of transgender phalloplasty and various urethroplasty surgeries for both cis-gender and transgender patients, reconstructive urologists often place both a UC and SP catheter intraoperatively, where the UC offers multiple functions. The UC serves to stent open a neourethral anastomosis, provide a platform for urethroplasty healing, and prevent any undrained urine from extravasating across the fresh anastomosis sutures [6]. After the UC is removed, the SP catheter is typically capped with a catheter plug so that the patient may begin to void per urethra, but the SP catheter will remain in place in case the patient is unable to void completely per urethra. Maximal bladder drainage is critical because urine accumulation increases risks of developing anastomotic strictures, fistulae, and infection. However, there is no consensus regarding how to maximize urinary drainage: by leaving one or both catheters open to drainage at all times? Nor is there consensus on which catheter, if either, drains the bladder more completely. With both a UC and SP catheter in place, many urologic surgeons today will cap one of the two catheters and leave the remaining catheter to drainage. In our institution’s transgender and genital reconstructive surgery program, per our routine postoperative pathway, both the UC and SP catheter are placed to gravity drainage (which, based on findings from Garcia et al., is most reliably achieved by placing the catheter drainage bag at floor-level to eliminate obstructive air-locks) [5]. The purpose of this study is to determine whether the UC or SP catheter consistently produces more urine output (UOP) than the other, by simultaneous dual gravity drainage. We hypothesized that the SP catheter would drain more urine relative to the UC.

## Methods

We retrospectively reviewed the UOP data from all inpatients who underwent Stage II Phalloplasty with urethral lengthening, including neourethroplasty, at a single institution from May 2017 to November 2020. In all cases, both a UC and SP catheter were placed intraoperatively. For the entire admission, all catheters were 16 French (Fr) and attached to drainage bags left on the ground without dependent loops to maximize gravity drainage. All catheters were consistently secured to the suprapubic region using a catheter stabilization adhesive clip device to standardize the connection location of the catheter and drainage bag while avoiding catheter traction or traumatic dislodgement with activity. Postoperatively, activity limitations included bedrest for approximately 48 hours, with transition to the upright seated position on postoperative day 3 followed by ambulation at least 3 times daily through to discharge. Patients were discharged with both catheters draining to gravity. UOP volume from each catheter was recorded separately, twice daily (daytime and nighttime shifts), through to the day of discharge. Given repeated measures of data, mixed model regression with an auto-regressive covariance structure was used to test for differences in UOP by catheter type (UC vs SP catheter), while covarying for both time of day (daytime: 7AM-7PM vs nighttime: 7PM-7AM) and activity status (Bedrest or Ambulatory Phases). The Bedrest Phase was defined as ranging from postoperative day 0 to 2 followed by the Ambulatory Phase which began on postoperative day 3 until time of discharge. UC and SP catheter output were evaluated during each phase of activity. As outliers were present, data was transformed with the Box-Cox method prior to analysis to meet assumptions necessary for parametric testing. The threshold for statistical significance was p<0.05. This study was approved by the Cedars-Sinai Medical Center Institutional Review Board (Pro #00055933).

## Results

A total of 14 transgender men were identified meeting inclusion criteria. There were no morbidly obese patients within the study population. All patients were without urinary catheters preoperatively. Mean length of stay was 4.4 days (range: 1.5, 7.0). The aggregate number of 12-hour shift UOP observations across all patients was 250 (UC 125 and SP catheter 125). The average total UOP per 12-hour shift for UC and SP catheters was 510 ml (SD: 437 ml) and 953 ml (SD: 769 ml), respectively. Table 1 illustrates a sample diary of UOP data for one inpatient admission.

Catheter output data for collective patients is summarized in Table 2. SP catheters had an estimated 410 ml higher output than UCs per 12-hour shift (p=0.002; 95% CI: 185, 636 ml). During daytime, SP catheters demonstrated higher UOP than UCs per 12-hour shift (Estimated Difference: 464 ml; p=0.002; 95% CI 211, 718 ml). During nighttime, a similar phenomenon was observed (Estimated Difference: 356 ml; p=0.009; 95% CI 104, 606 ml). When comparing mean UOP from each catheter during the Bedrest Phase, SP catheters averaged an estimated 295 ml higher UOP compared to UCs per 12-hour shift with a trend toward statistical significance (p=0.052; 95% CI −3, 594 ml). During the Ambulatory Phase, mean SP catheter UOP was an estimated 472 ml higher than UCs per 12-hour shift (p=0.009; 95% CI 142, 802 ml).

## Discussion

The key finding of the present study is that dual bladder drainage from indwelling catheters favors the SP catheter over the UC. On average, SP catheters drained 410 ml more urine than their UC counterpart across the entire postoperative course of stage II phalloplasty patients with urethral lengthening. This phenomenon of greater SP catheter drainage persisted during the Ambulatory Phase of the inpatient admission and regardless of time of day. During the Bedrest Phase, SP catheter UOP was again higher than UC UOP, however this result trended closely toward statistical significance (p=0.052). Nonetheless, these findings suggest that dual bladder drainage tends to favor the suprapubic outflow tract and this effect persists when ambulation is unrestricted.

This work is the first to compare urinary drainage efficacy among UCs and SP catheters simultaneously draining the same bladder. These findings are important, because in our experience many reconstructive urologists including gender affirming surgeons typically leave one of the two catheters to drainage and the other capped with a catheter plug. There are two theoretical benefits from capping one catheter and limiting urinary outflow to the other catheter/single drainage bag. First, there is a potentially lower likelihood of traumatic catheter dislodgment given the capped catheter is not connected to a drainage bag. This concern can be addressed through patient education of perioperative care, standardized nursing protocols for mobilizing patients, and use of skin adhesive catheter stabilization devices. Second, capping one of the two catheters simplifies postoperative care for the patient, as this allows the patient and nurses to manage only one catheter drainage bag, instead of two. However, we believe the presumed convenience of managing only one instead of two drainage bags is outweighed by the potential clinical benefit from maintaining both catheters to open gravity drainage. It is known that undrained urine can travel circumferentially along the outside of a UC, and especially so with the aid of uninhibited bladder contractions prompted by bladder fullness [5]. By maximizing bladder urinary drainage, we minimize the risk of exposing the fresh post-urethroplasty urethral anastomosis to urine and we aim to decrease the risk of catheter-related urinary tract infections, commonly associated with undrained bladder urine [7].

**Figure 1:**
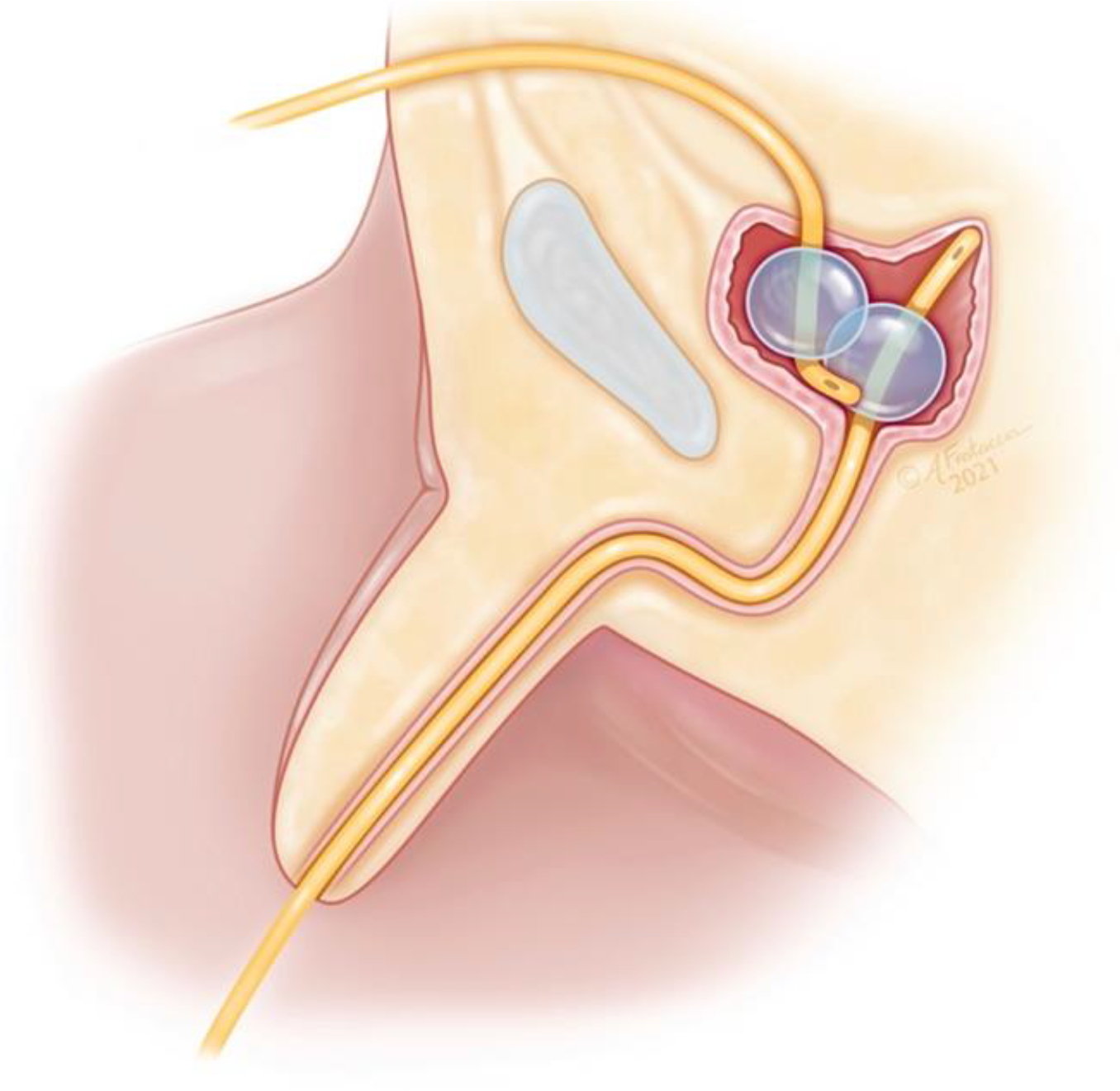
Anatomic Illustartion of Dual Bladder Driange using Urethral and Suprapubic Catheters Sagittal Section view of the decompressed Bladder Containing both an indwelling urethral catheter (UC) and suprapubic (SP) catheter, in a supine/seated position. The UC inlet abuts the bladder dome while the SP catheter inlet resides inferiorly at the bladder neck. The more gravity dependent location of the SP catheter inlet optimizes bladder driange.

The question remains: why does a SP catheter consistently drain the bladder more efficiently than a UC? We believe that the answer to this question is best explained by considering the location of each catheter’s drainage inlet within the bladder lumen. When a patient is in a supine position, the SP catheter passes through the suprapubic skin, posteriorly, deep into the bladder. The catheter is effectively positioned in a dorsal-to-ventral orientation relative to the bladder. Consequently, we hypothesize that the SP catheter tip and drainage inlet predominantly reside in a more gravity-dependent location, within the funnel-shaped bladder neck of the decompressed bladder (Figure 1). In contrast, the positioning of the UC within the bladder is angled in a net ventral-to-dorsal orientation, such that the UC’s tip and drainage inlet point upward towards the junction of the posterior bladder wall and bladder dome. At this location, when the patient is in a sitting or standing position, the UC drainage inlet likely resides anterior and superior to the bladder neck, where urine can pool. The UC tip can even potentially become buried within redundant bladder wall mucosa, obstructing its drainage of urine. When supine, it may appear counterintuitive that the SP catheter should drain more efficiently when it exits the body anteriorly, in the opposite direction of gravity. This can be explained by the Siphon Effect, a well-accepted principle in fluid dynamics [8,9]. In the case of the suprapubic catheter, atmospheric pressure drives fluid from the bladder against gravity to the apex of the catheter’s arc, and then gravitational force facilitates the remainder of drainage into the drainage bag. Each time the SP catheter drainage bag is emptied, the fluid column and meniscus remain at the apex of the catheter’s arc outside of the body, so that drainage continues immediately as urine enters the bladder. At any given time when the patient is supine, seated, or standing, the SP catheter drainage inlet likely has a more dependent location within the bladder than the UC as illustrated in figure 1.

While the utility of this study’s findings may at first appear to be restricted to reconstructive urologists, our results have broad applicability within urology, and in particular to general urology and pediatric urology. For instance, an alternative application of this drainage phenomenon involves the management of gross hematuria with continuous bladder irrigation (CBI), which has shown to minimize intravesical blood clot formation that can occlude an indwelling catheter resulting in clot urinary retention and potentially bladder rupture. CBI is typically performed with a single indwelling 3-way UC composed of an inflow channel, outflow channel, and a balloon port [10]. Suboptimal outflow will result in CBI occurring in a state of continuous bladder fullness/distension. A distended bladder may be at risk of rupture and in fact promote ongoing bleeding by precluding the bladder walls from compressing onto themselves as occurs when the bladder is empty. The objective with CBI is to maximize outlet drainage. Therefore, in patients with significant hematuria who happen to have both an indwelling UC and SP catheter of the same size, our results support use of the UC and SP catheter as the inflow and outflow channels, respectively, to optimize CBI drainage.

While there are surgical risks associated with SP catheter placement, the SP catheter permits better drainage while reducing risks of urinary tract infection, fistula formation, and urethral erosion associated with chronic indwelling UCs [11]. The risk of bowel injury with SP catheter placement can be further minimized by placing the patient in Trendelenburg positioning, maximally pre-filling the bladder before inserting the SP catheter trocar (unless contraindicated), and using image-guided techniques [12]. These findings further support the use of SP catheters over UCs in patients with a chronic neurogenic bladder due to spinal cord injury, who are sometimes managed long-term with a UC [11]. While no prior studies have compared simultaneous drainage from a UC and SP catheter draining the bladder, studies have reported improved benefits from the use of SP catheters relative to UCs. For instance, improved quality of life and cost-effectiveness has been seen using SP catheters instead of UCs after radical prostatectomy and stress urinary incontinence surgery [13,14] However, these studies did not include patients that simultaneously had both types of catheters.

Strengths of this study include the unique experimental design featuring UC and SP catheter drainage data from the same bladder, that allows for assessing relative drainage efficiency in the same urologic system in real-time. The data is further supported by a robust sample size of UOP sourced from 12-hour intervals via inpatient nursing shifts and the objective nature of UOP as the primary parameter of interest.

Limitations include the retrospective nature of the study’s data collection. Additionally, UOP documentation over 12 hours can be subject to variability based on inpatient factors such as amount of patient activity (i.e. time out of bed) and nurse or clinical assistant records. Importantly, these findings were observed with UC and SP catheters of the same size (16 Fr) and cannot be generalized to scenarios with discordant catheter sizes. While the study design distinguished between a Bedrest and Ambulatory Phase, the exact amount of ambulation may have varied across patients. Future studies evaluating catheter drainage may benefit from a larger patient population, however this statistical analysis performed was based on the discrete number of nursing shifts for which UOP was documented, totaling up to 125 unique 12-hour observation periods for each type of catheter.

The results of the present study ultimately validate our practice of leaving both the UC and SP catheter to gravity-dependent drainage to maximize bladder emptying among our postoperative patients given predominant outflow via the SP catheter. At our institution, we continue to place both the UC and SP catheter to gravity drainage in postoperative patients. Given that there is preferential drainage via suprapubic outflow in a system concurrently drained by two indwelling catheters (UC and SP catheter), an interesting question arises: is a solitary SP catheter able to accommodate more drainage in a bladder than a solitary UC? Given that there is preferential drainage via suprapubic outflow in a system concurrently drained by two indwelling catheters (UC and SP catheter), this finding allows us to postulate that the solitary SP catheter is likely to be more efficient and potentially accommodate more drainage in a bladder than a solitary UC. Future studies may consider assessing for differences in drainage using larger cohorts of patients involving control arms with solitary catheters (UC or SP catheters) and with catheters of varying diameters to evaluate postoperative outcomes and catheter drainage patterns while supine, seated, and standing for prolonged periods of time.

## Conclusions

Simultaneous bladder drainage with an indwelling UC and SP catheter is associated with greater and more efficient drainage from the SP catheter (35% vs 65%). When using two catheters, clinicians should consider placing both catheters to drainage in postoperative transgender phalloplasty patients and other reconstructive surgeries to maximize bladder drainage. These findings potentially suggest that when a single catheter must be relied upon for maximal bladder drainage, the SP catheter is potentially more likely to drain the bladder more completely and efficiently compared to a UC.

## Figures and Tables

**Table 1:** Sample Inpatient Urine Output Data by Postoperative Day in a Stage II Phalloplasty patient With Indwelling Urethral and Suprapubic Catheters Both to Gravity Driange

Catheter Size (French)	Total urine Output	Total %Urine Output	POD 1 AM	POD 1PM	POD 2AM	POD 2PM	POD 3AM	POD 3PM	POD 4AM
Urethral Catheter(16Fr):	2675 ml	31%	500 ml	325 ml	350 ml	200 ml	250 ml	400 ml	650 ml
Suprapubic Catheter (16 Fr):	5950 ml	69%	400 ml	450 ml	900 ml	1450 ml	600 ml	950 ml	1200 ml

(POD= Postoperative Day, AM = 7am to 7pm, PM = 7pm to 7am)

**Table 2:** Simultaneous Driange Pattern from Both Indwelling Urethral and Suprapubic Catheter (All Patient Urine Outputs Included n = Number of 12-hour Urine Output Measurements)

Urine Output	Urethral Catheter n= 125	Suprapublic Catheter n = 125	*P*-Value*	Estimated Difference* (95% Confidence Interval)
Mean Catheter Output	510±437 ml	953±769 ml	*p* = 0.002	410 ml (185, 636)
Daytime Output (7AM to 7PM)	536±479 ml	1044±828 ml	*p* = 0.002	464 ml (211, 718)
Nighttime Output (7PM to 7AM)	485±394 ml	863±701 ml	*p* = 0.009	356 ml (104, 606)
Mean Catheter Output - Bedrest Phase (Postop Day 0–2)	589±495 ml	919±795 ml	*p* = 0.052	295 ml (-3, 594)
Mean Catheter Output - Ambulatory Phase (Postop Day 3+)	411±327 ml	995±740 ml	*p* = 0.009	472 ml (142, 802)

* Computed from mixed model regeression

## List of abbreviations

CBI: Continuous Bladder Irrigation
Fr: French
SP: Suprapubic
UC: Urethral Catheter
UOP: Urine Output

## References

[1] Hanafy HM, Saad SM, & Al-Ghorab MM Ancient egyptian medicine Contribution to urology. Urology 4 (1974): 114–120. 21323001 10.1016/0090-4295(74)90124-1

[2] Feneley RC, Hopley IB, & Wells PN Urinary catheters: history, current status, adverse events and research agenda. Journal of medical engineering & technology 39 (2015): 459–470. 26383168 10.3109/03091902.2015.1085600 PMC4673556

[3] Foley F E A hemostatic bag catheter: a one piece latex rubber structure for control of bleeding and constant drainage following prostatic resection. The Journal of Urology 38 (1937): 134–139.

[4] Warren J W Catheter-associated urinary tract infections. International journal of antimicrobial agents 17 (2001): 299–303. 11295412 10.1016/s0924-8579(00)00359-9

[5] Garcia MM, Gulati S, Liepmann D Traditional Foley drainage systems-do they drain the bladder?. The Journal of urology 177 (2007): 203–207. 17162043 10.1016/j.juro.2006.08.101

[6] Falcone M, Timpano M, Oderda M. Suprapubic pedicled phalloplasty in transgender men: a multicentric retrospective cohort analysis. International journal of impotence research 33 (2020): 1–7. 10.1038/s41443-020-0238-4 32034312

[7] Gould CV, Umscheid CA, Agarwal RK. Guideline for prevention of catheter-associated urinary tract infections 2009. Infection Control & Hospital Epidemiology 31 (2010): 319–326. 10.1086/651091 20156062

[8] Potter A, Barnes FH. The siphon. Physics education 6 (1971): 362.

[9] Boatwright A, Hughes S, Barry J. The height limit of a siphon. Scientific reports 5 (2015): 1–8. 10.1038/srep16790 PMC4667279 26628323

[10] Willis GC, Tewelde SZ. The approach to the patient with hematuria. Emergency Medicine Clinics 37 (2019): 755–769. 31563206 10.1016/j.emc.2019.07.011

[11] Romo PGB, Smith CP, Cox A. Non-surgical urologic management of neurogenic bladder after spinal cord injury. World journal of urology 36 (2018): 1555–1568. 30051263 10.1007/s00345-018-2419-z

[12] Tompkins AJ, Travis M, Watne RE. Decreasing suprapubic tube-related injuries: results of case series and comprehensive literature review. Urologic nursing 34 (2014): 9–17. 24716375

[13] Orikasa S, Kanbe K, Shirai S. Suprapubic versus transurethral bladder drainage after radical prostatectomy: impact on patient discomfort. International Journal of Urology 19 (2012): 587–590. 22404531 10.1111/j.1442-2042.2012.02980.x

[14] Bergman A, Matthews L, Ballard CA. Suprapubic versus transurethral bladder drainage after surgery for stress urinary incontinence. Obstetrics and gynecology 69 (1987): 546–549. 3822295

